# *catena*-Poly[[[aqua­(pyrazino[2,3-*f*][1,10]phenanthroline)cadium(II)]-μ-4,4′-ethyl­enedibenzoato] *N*,*N*-dimethyl­formamide hemisolvate]

**DOI:** 10.1107/S1600536809024714

**Published:** 2009-07-04

**Authors:** Ya-Ping Li, Da-Jun Sun, Hu Zang, Li-Ying Han

**Affiliations:** a Department of Ophthalmology, the Second Hospital of Jilin University, Changchun 130041, People’s Republic of China; b Department of Vascular Surgery, the China–Japan Union Hospital of Jilin University, Changchun 130033, People’s Republic of China; c Department of Orthopedics, the China–Japan Union Hospital of Jilin University, Changchun 130033, People’s Republic of China; d Department of Gynaecology, the Second Hospital of Jilin University, Changchun 130041, People’s Republic of China

## Abstract

In the title compound, [Cd(C_16_H_10_O_4_)(C_14_H_8_N_4_)(H_2_O)]·0.5C_3_H_7_NO, the Cd^II^ atom is six-coordinated by two N atoms from one pyrazino[2,3-*f*][1,10]phenanthroline ligand, three carboxyl­ate O atoms from two different 4,4′-ethyl­enedibenzoate ligands, and one water mol­ecule in a distorted octa­hedral environment. The two 4,4′-ethyl­enedibenzoate dianions are located on inversion centres bridging two neighboring Cd^II^ centres. O—H⋯O hydrogen-bonding inter­actions further stabilize the crystal structure.  The DMF molecule is equally disordered about a center of inversion.

## Related literature

For general background and related structures, see: Wang *et al.* (2008 ▶); Yang *et al.* (2007 ▶); Batten & Robson (1998 ▶); Qiao *et al.* (2008 ▶).
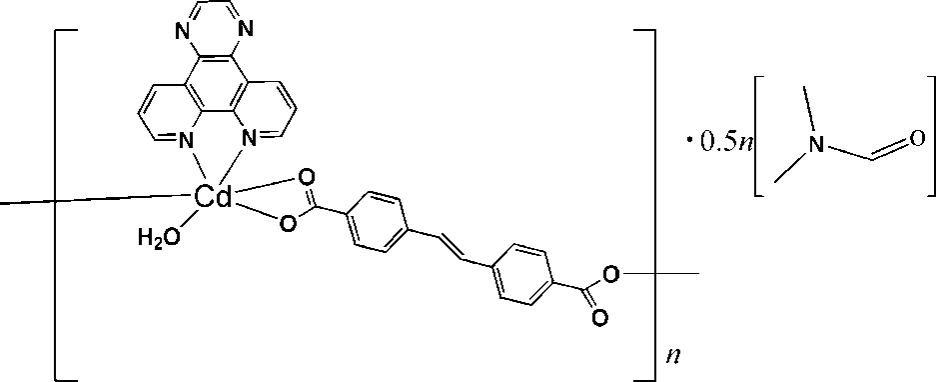


## Experimental

### 

#### Crystal data

[Cd(C_16_H_10_O_4_)(C_14_H_8_N_4_)(H_2_O)]·0.5C_3_H_7_NO
*M* *_r_* = 665.45
Triclinic, 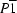

*a* = 11.4348 (5) Å
*b* = 11.5167 (4) Å
*c* = 11.7530 (5) Å
α = 84.654 (3)°
β = 69.154 (4)°
γ = 84.027 (3)°
*V* = 1435.97 (10) Å^3^
*Z* = 2
Mo *K*α radiation
μ = 0.81 mm^−1^
*T* = 293 K
0.30 × 0.28 × 0.16 mm

#### Data collection

Bruker SMART APEX diffractometer
Absorption correction: multi-scan (*SADABS*; Sheldrick, 1996 ▶) *T*_min_ = 0.778, *T*_max_ = 0.801 (expected range = 0.853–0.878)
14619 measured reflections
5828 independent reflections
3930 reflections with *I* > 2σ(*I*)
*R*_int_ = 0.047

#### Refinement

*R*[*F* ^2^ > 2σ(*F* ^2^)] = 0.035
*wR*(*F* ^2^) = 0.056
*S* = 0.86
5828 reflections
416 parameters
33 restraints
H atoms treated by a mixture of independent and constrained refinement
Δρ_max_ = 0.46 e Å^−3^
Δρ_min_ = −0.32 e Å^−3^

### 

Data collection: *SMART* (Bruker, 1997 ▶); cell refinement: *SAINT* (Bruker, 1999 ▶); data reduction: *SAINT*; program(s) used to solve structure: *SHELXS97* (Sheldrick, 2008 ▶); program(s) used to refine structure: *SHELXL97* (Sheldrick, 2008 ▶); molecular graphics: *DIAMOND* (Brandenburg, 2006 ▶); software used to prepare material for publication: *SHELXL97*.

## Supplementary Material

Crystal structure: contains datablocks global, I. DOI: 10.1107/S1600536809024714/bt2984sup1.cif

Structure factors: contains datablocks I. DOI: 10.1107/S1600536809024714/bt2984Isup2.hkl

Additional supplementary materials:  crystallographic information; 3D view; checkCIF report

## Figures and Tables

**Table 1:** Hydrogen-bond geometry (Å, °)

*D*—H⋯*A*	*D*—H	H⋯*A*	*D*⋯*A*	*D*—H⋯*A*
O5—H5*A*⋯O2	0.844 (10)	1.854 (13)	2.655 (3)	158 (3)
O5—H5*B*⋯O4^i^	0.843 (10)	1.881 (12)	2.713 (3)	169 (3)

Symmetry code: (i) 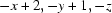.

**Fig. 1.**
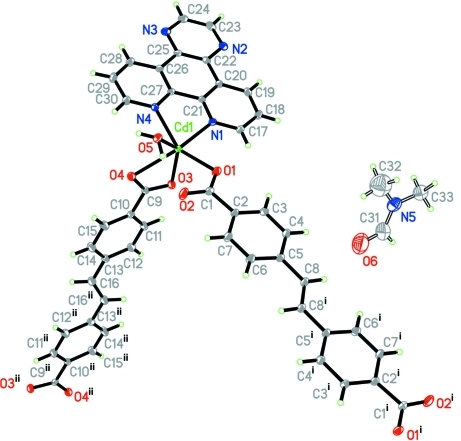
Perspective view of the title compound, showing the atomic numbering scheme. Displacement ellipsoids are drawn at the 20% probability level (symmetry operations i: 3 - x, -y, 1 - z; ii: 3 - x, 2 - y, -z).

**Fig. 2.**
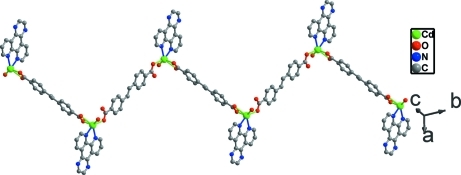
View of the zigzag chain of the title compound.

## supplementary crystallographic information

### Comment

Current interest in polymeric coordination networks is rapidly expanding not only for their potential applications in host–guest chemistry, ion exchange, gas storage, and nonlinear optics, but also for their intriguing variety of topologies (Batten & Robson, 1998). In this regard, chain structures have received much attention in coordination chemistry and materials chemistry. An appropriate flexible bidentate organic acid bridge could be useful in the formation of chains in the presence of secondary ligands, such as 2,2'-bipyridine (bipy) and 1,10-phenanthroline (phen) (Wang *et al.*, 2008). The N atoms from the secondary ligand may occupy two coordination positions of the metal ions. The rest of the coordination positions are available for other carboxylate ligands to allow the formation of a chain. The use of aromatic carboxylic acids in the syntheses of chain coordination polymers has aroused enormous interest owing to their versatile coordination modes and varieties of structural conformations. So far, aromatic multicarboxylate ligands, such as 1,2-benzenedicarboxylic acid, 1,3-benzenedicarboxylic acid and 1,4-benzenedicarboxylic acid, have been widely used to construct chain structures with interesting properties (Qiao *et al.*, 2008). In this regard, 4,4'-ethylenedibenzoatic acid (H_2_bpea) is also a good ligand in coordination chemistry because of its strong coordination ability and versatile coordination modes, so much attention has been paid to it in recent decades. On the other hand, the phen molecule, as one type of important organic ligand, has been widely utilized in the construction of chain structure complexes. An important derivative of phen, pyrazino[2,3-*f*][1,10]phenanthroline (pyphen) has been widely used to synthesize various Ru(II) complexes in order to recognize the secondary structure of DNA (Qiao *et al.*, 2008). However, the complexes constructed by carboxylate ligand and pyphen ligand have rarely been documented (Yang *et al.*, 2007). In the present study, we selected H_2_bpea as a linker and pyphen as a secondary chelating ligand, forming a unique zigzag chain coordination polymer [Cd(pyphen)(bpea)(H_2_O)]^.^0.5DMF (DMF = dimethylformamide).

As shown in Fig. 1, each Cd^II^ atom is six-coordinated by two N atoms from one pyphen, three carboxylate O atoms from two different bpea ligands, and one water molecule in a distorted octahedral sphere. The two bpea dianions are situated across inversion centres. The bpea dianions bridge two neighboring Cd^II^ centres, yielding a one-dimensional chain (Fig. 2). The *O*—*H*···O_carboxylate_ H-bonding interactions further stabilize the structure of (I) (Table 1).

### Experimental

A mixture of 4,4'-ethylenedibenzoatic acid (0.5 mmol), pyrazino[2,3-*f*][1,10]phenanthroline (0.5 mmol), NaOH (1 mmol) and CdCl_2_^.^2H_2_O (0.5 mmol) was suspended in deionized water (12 ml) and sealed in a 20-ml Teflon-lined autoclave. Upon heating at 433 K for one week, the autoclave was slowly cooled to room temperature. The crystals were collected, washed with deionized water, and dried.

### Refinement

H atoms bonded to C were positioned geometrically (C—H = 0.93 Å) and refined as riding, with *U*_iso_(H)=1.2*U*_eq_(C). The water H atoms were located in a difference Fourier map, and were refined with distance restraints of O–H 0.85±0.01 and H···H 1.39±0.01 Å. Their displacement parameters were freely refined.

The DMF molecule   is disordered about a center-of-inversion. The C–O length was restrained to 1.25±0.01 Å; the *N*–*C*(carbonyl) length was restrained to 1.35±0.01 Å and the oher two N–C lengths to 1.45±0.01 Å. The non-H atoms were restrained to lie on a common plane. The anisotropic temperature factors were restrained to be nearly isotropic.

### Crystal data

**Table:**

[Cd(C_16_H_10_O_4_)(C_14_H_8_N_4_)(H_2_O)]·0.5C_3_H_7_NO	*Z* = 2
*M**_r_* = 665.45	*F*(000) = 672
Triclinic, *P*1	*D*_x_ = 1.539 Mg m^−^^3^
Hall symbol: -P 1	Mo *K*α radiation, λ = 0.71073 Å
*a* = 11.4348 (5) Å	Cell parameters from 5828 reflections
*b* = 11.5167 (4) Å	θ = 3.1–26.4°
*c* = 11.7530 (5) Å	µ = 0.81 mm^−^^1^
α = 84.654 (3)°	*T* = 293 K
β = 69.154 (4)°	Block, pale yellow
γ = 84.027 (3)°	0.30 × 0.28 × 0.16 mm
*V* = 1435.97 (10) Å^3^	

### Data collection

**Table:**

Bruker SMART APEX diffractometer	5828 independent reflections
Radiation source: fine-focus sealed tube	3930 reflections with *I* > 2σ(*I*)
graphite	*R*_int_ = 0.047
φ and ω scans	θ_max_ = 26.4°, θ_min_ = 4.3°
Absorption correction: multi-scan (*SADABS*; Sheldrick, 1996)	*h* = −14→14
*T*_min_ = 0.778, *T*_max_ = 0.801	*k* = −14→14
14619 measured reflections	*l* = −14→10

### Refinement

**Table:**

Refinement on *F*^2^	Primary atom site location: structure-invariant direct methods
Least-squares matrix: full	Secondary atom site location: difference Fourier map
*R*[*F*^2^ > 2σ(*F*^2^)] = 0.035	Hydrogen site location: inferred from neighbouring sites
*wR*(*F*^2^) = 0.056	H atoms treated by a mixture of independent and constrained refinement
*S* = 0.86	*w* = 1/[σ^2^(*F*_o_^2^) + (0.0206*P*)^2^] where *P* = (*F*_o_^2^ + 2*F*_c_^2^)/3
5828 reflections	(Δ/σ)_max_ = 0.001
416 parameters	Δρ_max_ = 0.46 e Å^−^^3^
33 restraints	Δρ_min_ = −0.32 e Å^−^^3^

### Special details

**Table:**

Geometry. All e.s.d.'s (except the e.s.d. in the dihedral angle between two l.s. planes) are estimated using the full covariance matrix. The cell e.s.d.'s are taken into account individually in the estimation of e.s.d.'s in distances, angles and torsion angles; correlations between e.s.d.'s in cell parameters are only used when they are defined by crystal symmetry. An approximate (isotropic) treatment of cell e.s.d.'s is used for estimating e.s.d.'s involving l.s. planes.
Refinement. Refinement of *F*^2^ against ALL reflections. The weighted *R*-factor *wR* and goodness of fit *S* are based on *F*^2^, conventional *R*-factors *R* are based on *F*, with *F* set to zero for negative *F*^2^. The threshold expression of *F*^2^ > σ(*F*^2^) is used only for calculating *R*-factors(gt) *etc*. and is not relevant to the choice of reflections for refinement. *R*-factors based on *F*^2^ are statistically about twice as large as those based on *F*, and *R*- factors based on ALL data will be even larger.

### Fractional atomic coordinates and isotropic or equivalent isotropic displacement parameters (Å^2^)

**Table:**

	*x*	*y*	*z*	*U*_iso_*/*U*_eq_	Occ. (<1)
Cd1	0.930477 (18)	0.475704 (19)	0.26922 (2)	0.03672 (8)	
O1	1.01917 (18)	0.33906 (17)	0.36539 (18)	0.0514 (5)	
O2	1.1531 (2)	0.2867 (2)	0.1867 (2)	0.0900 (9)	
O3	1.04743 (18)	0.62445 (17)	0.27400 (18)	0.0524 (5)	
O4	1.06257 (16)	0.58849 (16)	0.08946 (17)	0.0457 (5)	
O5	0.94576 (19)	0.33849 (18)	0.13394 (19)	0.0488 (5)	
N1	0.77160 (18)	0.51028 (17)	0.45663 (19)	0.0347 (5)	
N2	0.3668 (2)	0.7125 (2)	0.6508 (2)	0.0472 (6)	
N3	0.3523 (2)	0.7770 (2)	0.4180 (2)	0.0495 (6)	
N4	0.75597 (19)	0.57195 (19)	0.2329 (2)	0.0389 (5)	
C1	1.1176 (3)	0.2852 (3)	0.2991 (3)	0.0479 (8)	
C2	1.1979 (2)	0.2147 (2)	0.3624 (3)	0.0388 (7)	
C3	1.1667 (3)	0.2076 (2)	0.4866 (3)	0.0441 (7)	
H3	1.0915	0.2444	0.5351	0.053*	
C4	1.2459 (3)	0.1462 (2)	0.5410 (3)	0.0444 (7)	
H4	1.2235	0.1429	0.6254	0.053*	
C5	1.3589 (2)	0.0892 (2)	0.4706 (3)	0.0373 (7)	
C6	1.3878 (3)	0.0959 (2)	0.3464 (3)	0.0478 (8)	
H6	1.4619	0.0576	0.2976	0.057*	
C7	1.3099 (3)	0.1580 (2)	0.2922 (3)	0.0491 (8)	
H7	1.3327	0.1620	0.2077	0.059*	
C8	1.4441 (2)	0.0270 (2)	0.5287 (3)	0.0420 (7)	
H8	1.4176	0.0253	0.6134	0.050*	
C9	1.0938 (2)	0.6436 (2)	0.1608 (3)	0.0372 (7)	
C10	1.1894 (2)	0.7311 (2)	0.1113 (3)	0.0388 (7)	
C11	1.2109 (3)	0.8027 (3)	0.1886 (3)	0.0508 (8)	
H11	1.1647	0.7962	0.2716	0.061*	
C12	1.2997 (3)	0.8839 (3)	0.1446 (3)	0.0521 (8)	
H12	1.3121	0.9318	0.1982	0.063*	
C13	1.3703 (3)	0.8948 (3)	0.0222 (3)	0.0477 (8)	
C14	1.3477 (3)	0.8228 (3)	−0.0547 (3)	0.0625 (9)	
H14	1.3931	0.8295	−0.1379	0.075*	
C15	1.2593 (3)	0.7415 (3)	−0.0101 (3)	0.0583 (9)	
H15	1.2471	0.6932	−0.0634	0.070*	
C16	1.4677 (3)	0.9788 (3)	−0.0278 (3)	0.0557 (8)	
H16	1.4851	1.0047	−0.1090	0.067*	
C17	0.7813 (3)	0.4793 (2)	0.5637 (3)	0.0411 (7)	
H17	0.8532	0.4349	0.5663	0.049*	
C18	0.6894 (3)	0.5096 (3)	0.6725 (3)	0.0495 (8)	
H18	0.6994	0.4860	0.7465	0.059*	
C19	0.5837 (3)	0.5748 (2)	0.6695 (3)	0.0460 (7)	
H19	0.5207	0.5957	0.7418	0.055*	
C20	0.5706 (2)	0.6099 (2)	0.5580 (2)	0.0358 (6)	
C21	0.6675 (2)	0.5753 (2)	0.4523 (2)	0.0317 (6)	
C22	0.4609 (2)	0.6801 (2)	0.5460 (3)	0.0378 (7)	
C23	0.2712 (3)	0.7744 (3)	0.6344 (3)	0.0588 (9)	
H23	0.2047	0.7987	0.7027	0.071*	
C24	0.2637 (3)	0.8057 (3)	0.5203 (3)	0.0553 (9)	
H24	0.1921	0.8494	0.5162	0.066*	
C25	0.4540 (2)	0.7124 (2)	0.4320 (3)	0.0376 (7)	
C26	0.5547 (2)	0.6766 (2)	0.3223 (3)	0.0375 (7)	
C27	0.6604 (2)	0.6090 (2)	0.3322 (2)	0.0335 (6)	
C28	0.5517 (3)	0.7060 (3)	0.2057 (3)	0.0547 (8)	
H28	0.4835	0.7516	0.1956	0.066*	
C29	0.6494 (3)	0.6677 (3)	0.1055 (3)	0.0624 (9)	
H29	0.6483	0.6861	0.0272	0.075*	
C30	0.7495 (3)	0.6011 (3)	0.1242 (3)	0.0533 (8)	
H30	0.8158	0.5754	0.0564	0.064*	
O6	1.2305 (11)	0.0866 (9)	0.8336 (11)	0.226 (5)	0.50
N5	1.0516 (10)	0.0318 (7)	0.9847 (10)	0.114 (3)	0.50
C31	1.1577 (12)	0.0819 (9)	0.9420 (12)	0.158 (5)	0.50
H31	1.1810	0.1175	0.9977	0.190*	0.50
C32	1.0130 (11)	−0.0250 (9)	0.8965 (10)	0.109 (5)	0.50
H32A	1.0591	0.0037	0.8150	0.164*	0.50
H32B	0.9248	−0.0073	0.9133	0.164*	0.50
H32C	1.0303	−0.1082	0.9042	0.164*	0.50
C33	0.969 (2)	0.0280 (19)	1.1125 (16)	0.35 (2)	0.50
H33A	1.0121	0.0548	1.1612	0.523*	0.50
H33B	0.9481	−0.0508	1.1393	0.523*	0.50
H33C	0.8944	0.0777	1.1211	0.523*	0.50
H5A	1.0175 (16)	0.312 (3)	0.134 (3)	0.085 (13)*	
H5B	0.944 (3)	0.352 (3)	0.0627 (14)	0.083 (13)*	

### Atomic displacement parameters (Å^2^)

**Table:**

	*U*^11^	*U*^22^	*U*^33^	*U*^12^	*U*^13^	*U*^23^
Cd1	0.03009 (12)	0.03969 (13)	0.04059 (13)	−0.00105 (8)	−0.01371 (9)	0.00032 (9)
O1	0.0430 (12)	0.0577 (13)	0.0521 (13)	0.0151 (10)	−0.0206 (11)	−0.0016 (11)
O2	0.0628 (15)	0.150 (2)	0.0472 (16)	0.0408 (15)	−0.0228 (13)	0.0055 (15)
O3	0.0556 (13)	0.0691 (14)	0.0373 (13)	−0.0267 (10)	−0.0190 (10)	0.0089 (10)
O4	0.0500 (12)	0.0505 (12)	0.0401 (12)	−0.0199 (10)	−0.0162 (10)	−0.0002 (10)
O5	0.0511 (14)	0.0560 (14)	0.0458 (14)	−0.0062 (11)	−0.0242 (11)	−0.0029 (11)
N1	0.0326 (13)	0.0345 (13)	0.0399 (14)	−0.0057 (10)	−0.0157 (11)	0.0003 (10)
N2	0.0421 (15)	0.0447 (15)	0.0472 (16)	−0.0022 (12)	−0.0063 (13)	−0.0044 (12)
N3	0.0364 (14)	0.0457 (15)	0.0652 (18)	0.0036 (12)	−0.0176 (14)	−0.0050 (13)
N4	0.0318 (13)	0.0485 (15)	0.0339 (14)	0.0001 (11)	−0.0103 (11)	0.0016 (11)
C1	0.0404 (19)	0.056 (2)	0.050 (2)	0.0040 (16)	−0.0224 (17)	0.0034 (16)
C2	0.0393 (17)	0.0340 (16)	0.0466 (19)	0.0001 (13)	−0.0211 (15)	0.0018 (13)
C3	0.0351 (16)	0.0457 (18)	0.049 (2)	0.0063 (13)	−0.0146 (15)	−0.0013 (15)
C4	0.0460 (18)	0.0463 (18)	0.0420 (18)	−0.0004 (14)	−0.0187 (15)	0.0008 (14)
C5	0.0386 (16)	0.0303 (15)	0.0479 (19)	−0.0004 (13)	−0.0226 (15)	0.0007 (13)
C6	0.0413 (17)	0.0522 (19)	0.048 (2)	0.0141 (14)	−0.0171 (15)	−0.0078 (15)
C7	0.0496 (19)	0.056 (2)	0.0414 (18)	0.0104 (16)	−0.0202 (16)	−0.0015 (15)
C8	0.0468 (17)	0.0392 (18)	0.0450 (18)	−0.0010 (14)	−0.0243 (15)	0.0029 (14)
C9	0.0353 (16)	0.0387 (17)	0.0393 (18)	−0.0025 (13)	−0.0163 (14)	0.0014 (14)
C10	0.0373 (16)	0.0454 (18)	0.0372 (17)	−0.0098 (13)	−0.0165 (15)	0.0014 (14)
C11	0.0504 (19)	0.062 (2)	0.0394 (18)	−0.0161 (16)	−0.0134 (15)	0.0024 (16)
C12	0.0519 (19)	0.061 (2)	0.047 (2)	−0.0169 (17)	−0.0178 (17)	−0.0055 (16)
C13	0.0421 (18)	0.053 (2)	0.048 (2)	−0.0132 (15)	−0.0154 (16)	0.0050 (16)
C14	0.063 (2)	0.083 (3)	0.0411 (19)	−0.0369 (19)	−0.0091 (17)	−0.0048 (18)
C15	0.058 (2)	0.068 (2)	0.051 (2)	−0.0239 (18)	−0.0148 (18)	−0.0103 (17)
C16	0.059 (2)	0.065 (2)	0.044 (2)	−0.0200 (17)	−0.0156 (15)	0.0007 (16)
C17	0.0441 (17)	0.0397 (17)	0.0461 (19)	−0.0009 (14)	−0.0252 (16)	0.0001 (14)
C18	0.058 (2)	0.058 (2)	0.0383 (19)	−0.0077 (17)	−0.0253 (17)	0.0011 (15)
C19	0.0466 (18)	0.0502 (19)	0.0364 (18)	−0.0100 (15)	−0.0068 (15)	−0.0033 (14)
C20	0.0371 (16)	0.0332 (16)	0.0359 (17)	−0.0075 (13)	−0.0102 (14)	−0.0004 (13)
C21	0.0304 (15)	0.0281 (14)	0.0400 (17)	−0.0076 (12)	−0.0161 (13)	0.0028 (12)
C22	0.0349 (16)	0.0313 (16)	0.0449 (18)	−0.0091 (13)	−0.0084 (14)	−0.0058 (13)
C23	0.0406 (19)	0.051 (2)	0.067 (3)	0.0019 (16)	0.0037 (17)	−0.0104 (18)
C24	0.0383 (19)	0.049 (2)	0.076 (3)	0.0022 (15)	−0.0183 (19)	−0.0041 (18)
C25	0.0282 (15)	0.0355 (16)	0.0485 (19)	−0.0032 (13)	−0.0120 (14)	−0.0044 (14)
C26	0.0322 (15)	0.0382 (16)	0.0427 (18)	−0.0033 (13)	−0.0147 (14)	0.0017 (13)
C27	0.0290 (15)	0.0317 (15)	0.0412 (17)	−0.0032 (12)	−0.0136 (14)	−0.0027 (13)
C28	0.0457 (19)	0.066 (2)	0.054 (2)	0.0096 (16)	−0.0237 (17)	0.0025 (17)
C29	0.056 (2)	0.090 (3)	0.0390 (19)	0.0112 (19)	−0.0206 (17)	0.0050 (18)
C30	0.0432 (18)	0.072 (2)	0.0390 (19)	0.0051 (16)	−0.0101 (15)	−0.0017 (16)
O6	0.254 (8)	0.250 (8)	0.182 (8)	−0.014 (7)	−0.095 (7)	0.010 (7)
N5	0.108 (8)	0.097 (7)	0.144 (9)	0.035 (5)	−0.057 (8)	−0.028 (7)
C31	0.185 (9)	0.164 (9)	0.127 (8)	−0.004 (8)	−0.059 (7)	−0.004 (7)
C32	0.103 (7)	0.079 (6)	0.123 (8)	0.035 (5)	−0.022 (6)	−0.016 (5)
C33	0.35 (2)	0.34 (2)	0.35 (3)	−0.003 (10)	−0.117 (12)	−0.023 (10)

### Geometric parameters (Å, °)

**Table:**

Cd1—O1	2.2215 (18)	C12—H12	0.9300
Cd1—O5	2.295 (2)	C13—C14	1.385 (4)
Cd1—O3	2.2966 (18)	C13—C16	1.475 (4)
Cd1—N4	2.335 (2)	C14—C15	1.379 (4)
Cd1—N1	2.340 (2)	C14—H14	0.9300
Cd1—O4	2.4613 (18)	C15—H15	0.9300
Cd1—C9	2.729 (3)	C16—C16^ii^	1.298 (5)
O1—C1	1.256 (3)	C16—H16	0.9300
O2—C1	1.234 (3)	C17—C18	1.383 (4)
O3—C9	1.252 (3)	C17—H17	0.9300
O4—C9	1.262 (3)	C18—C19	1.364 (4)
O5—H5A	0.844 (10)	C18—H18	0.9300
O5—H5B	0.843 (10)	C19—C20	1.390 (4)
N1—C17	1.316 (3)	C19—H19	0.9300
N1—C21	1.353 (3)	C20—C21	1.396 (4)
N2—C23	1.305 (4)	C20—C22	1.463 (4)
N2—C22	1.369 (3)	C21—C27	1.457 (3)
N3—C24	1.311 (4)	C22—C25	1.385 (4)
N3—C25	1.367 (3)	C23—C24	1.385 (4)
N4—C30	1.315 (3)	C23—H23	0.9300
N4—C27	1.352 (3)	C24—H24	0.9300
C1—C2	1.509 (4)	C25—C26	1.449 (4)
C2—C3	1.371 (4)	C26—C28	1.392 (4)
C2—C7	1.386 (4)	C26—C27	1.403 (3)
C3—C4	1.386 (4)	C28—C29	1.374 (4)
C3—H3	0.9300	C28—H28	0.9300
C4—C5	1.396 (4)	C29—C30	1.383 (4)
C4—H4	0.9300	C29—H29	0.9300
C5—C6	1.374 (4)	C30—H30	0.9300
C5—C8	1.472 (3)	O6—C31	1.249 (9)
C6—C7	1.377 (4)	N5—C31	1.310 (9)
C6—H6	0.9300	N5—C33	1.461 (10)
C7—H7	0.9300	N5—C32	1.486 (9)
C8—C8^i^	1.332 (5)	C31—H31	0.9300
C8—H8	0.9300	C32—H32A	0.9600
C9—C10	1.489 (4)	C32—H32B	0.9600
C10—C15	1.366 (4)	C32—H32C	0.9600
C10—C11	1.381 (4)	C33—H33A	0.9600
C11—C12	1.381 (4)	C33—H33B	0.9600
C11—H11	0.9300	C33—H33C	0.9600
C12—C13	1.378 (4)		
			
O1—Cd1—O5	87.36 (7)	C11—C10—C9	120.2 (3)
O1—Cd1—O3	96.57 (7)	C10—C11—C12	121.1 (3)
O5—Cd1—O3	135.60 (7)	C10—C11—H11	119.4
O1—Cd1—N4	152.12 (8)	C12—C11—H11	119.4
O5—Cd1—N4	90.76 (8)	C13—C12—C11	120.7 (3)
O3—Cd1—N4	104.04 (7)	C13—C12—H12	119.7
O1—Cd1—N1	88.42 (7)	C11—C12—H12	119.7
O5—Cd1—N1	128.74 (7)	C12—C13—C14	117.8 (3)
O3—Cd1—N1	95.62 (7)	C12—C13—C16	122.3 (3)
N4—Cd1—N1	71.24 (7)	C14—C13—C16	120.0 (3)
O1—Cd1—O4	119.82 (7)	C15—C14—C13	121.2 (3)
O5—Cd1—O4	85.14 (7)	C15—C14—H14	119.4
O3—Cd1—O4	54.65 (6)	C13—C14—H14	119.4
N4—Cd1—O4	87.67 (7)	C10—C15—C14	120.8 (3)
N1—Cd1—O4	138.68 (7)	C10—C15—H15	119.6
O1—Cd1—C9	109.35 (7)	C14—C15—H15	119.6
O5—Cd1—C9	110.65 (8)	C16^ii^—C16—C13	128.0 (4)
O3—Cd1—C9	27.14 (7)	C16^ii^—C16—H16	116.0
N4—Cd1—C9	97.33 (8)	C13—C16—H16	116.0
N1—Cd1—C9	118.85 (8)	N1—C17—C18	122.9 (2)
O4—Cd1—C9	27.52 (7)	N1—C17—H17	118.5
C1—O1—Cd1	115.60 (19)	C18—C17—H17	118.5
C9—O3—Cd1	96.06 (16)	C19—C18—C17	118.9 (3)
C9—O4—Cd1	88.15 (16)	C19—C18—H18	120.5
Cd1—O5—H5A	92 (2)	C17—C18—H18	120.5
Cd1—O5—H5B	127 (2)	C18—C19—C20	119.7 (3)
H5A—O5—H5B	112.0 (17)	C18—C19—H19	120.1
C17—N1—C21	118.8 (2)	C20—C19—H19	120.1
C17—N1—Cd1	124.57 (17)	C19—C20—C21	117.8 (2)
C21—N1—Cd1	116.43 (16)	C19—C20—C22	123.5 (3)
C23—N2—C22	114.9 (3)	C21—C20—C22	118.7 (2)
C24—N3—C25	114.6 (3)	N1—C21—C20	121.8 (2)
C30—N4—C27	118.8 (2)	N1—C21—C27	117.3 (2)
C30—N4—Cd1	124.95 (18)	C20—C21—C27	120.9 (2)
C27—N4—Cd1	115.94 (16)	N2—C22—C25	121.8 (2)
O2—C1—O1	124.8 (3)	N2—C22—C20	117.7 (2)
O2—C1—C2	118.1 (3)	C25—C22—C20	120.5 (2)
O1—C1—C2	117.0 (3)	N2—C23—C24	123.4 (3)
C3—C2—C7	118.5 (2)	N2—C23—H23	118.3
C3—C2—C1	122.6 (3)	C24—C23—H23	118.3
C7—C2—C1	118.8 (3)	N3—C24—C23	123.5 (3)
C2—C3—C4	120.9 (3)	N3—C24—H24	118.2
C2—C3—H3	119.6	C23—C24—H24	118.2
C4—C3—H3	119.6	N3—C25—C22	121.8 (3)
C3—C4—C5	120.8 (3)	N3—C25—C26	117.4 (2)
C3—C4—H4	119.6	C22—C25—C26	120.8 (2)
C5—C4—H4	119.6	C28—C26—C27	117.7 (3)
C6—C5—C4	117.5 (2)	C28—C26—C25	122.9 (2)
C6—C5—C8	122.0 (2)	C27—C26—C25	119.4 (2)
C4—C5—C8	120.5 (3)	N4—C27—C26	121.7 (2)
C5—C6—C7	121.7 (3)	N4—C27—C21	118.5 (2)
C5—C6—H6	119.1	C26—C27—C21	119.8 (2)
C7—C6—H6	119.1	C29—C28—C26	119.9 (3)
C6—C7—C2	120.6 (3)	C29—C28—H28	120.0
C6—C7—H7	119.7	C26—C28—H28	120.0
C2—C7—H7	119.7	C28—C29—C30	118.3 (3)
C8^i^—C8—C5	126.0 (3)	C28—C29—H29	120.8
C8^i^—C8—H8	117.0	C30—C29—H29	120.8
C5—C8—H8	117.0	N4—C30—C29	123.5 (3)
O3—C9—O4	121.1 (3)	N4—C30—H30	118.2
O3—C9—C10	118.6 (2)	C29—C30—H30	118.2
O4—C9—C10	120.3 (3)	C31—N5—C33	124.4 (15)
O3—C9—Cd1	56.80 (14)	C31—N5—C32	117.4 (13)
O4—C9—Cd1	64.33 (14)	C33—N5—C32	118.2 (18)
C10—C9—Cd1	173.92 (18)	O6—C31—N5	126.3 (15)
C15—C10—C11	118.3 (3)	O6—C31—H31	116.9
C15—C10—C9	121.5 (3)	N5—C31—H31	116.9
			
O5—Cd1—O1—C1	49.6 (2)	N4—Cd1—C9—C10	148.5 (18)
O3—Cd1—O1—C1	−86.0 (2)	N1—Cd1—C9—C10	75.9 (19)
N4—Cd1—O1—C1	136.2 (2)	O4—Cd1—C9—C10	−141.1 (19)
N1—Cd1—O1—C1	178.5 (2)	O3—C9—C10—C15	168.5 (3)
O4—Cd1—O1—C1	−33.3 (2)	O4—C9—C10—C15	−10.1 (4)
C9—Cd1—O1—C1	−61.4 (2)	Cd1—C9—C10—C15	128.9 (18)
O1—Cd1—O3—C9	120.41 (16)	O3—C9—C10—C11	−10.5 (4)
O5—Cd1—O3—C9	27.5 (2)	O4—C9—C10—C11	171.0 (2)
N4—Cd1—O3—C9	−78.50 (16)	Cd1—C9—C10—C11	−50 (2)
N1—Cd1—O3—C9	−150.53 (16)	C15—C10—C11—C12	0.6 (4)
O4—Cd1—O3—C9	−1.73 (14)	C9—C10—C11—C12	179.6 (3)
O1—Cd1—O4—C9	−74.12 (16)	C10—C11—C12—C13	−0.6 (4)
O5—Cd1—O4—C9	−158.27 (15)	C11—C12—C13—C14	0.8 (4)
O3—Cd1—O4—C9	1.70 (14)	C11—C12—C13—C16	−178.8 (3)
N4—Cd1—O4—C9	110.77 (15)	C12—C13—C14—C15	−1.2 (5)
N1—Cd1—O4—C9	53.02 (18)	C16—C13—C14—C15	178.5 (3)
O1—Cd1—N1—C17	19.2 (2)	C11—C10—C15—C14	−0.9 (5)
O5—Cd1—N1—C17	104.5 (2)	C9—C10—C15—C14	−179.9 (3)
O3—Cd1—N1—C17	−77.3 (2)	C13—C14—C15—C10	1.3 (5)
N4—Cd1—N1—C17	179.8 (2)	C12—C13—C16—C16^ii^	26.4 (6)
O4—Cd1—N1—C17	−117.1 (2)	C14—C13—C16—C16^ii^	−153.2 (4)
C9—Cd1—N1—C17	−92.1 (2)	C21—N1—C17—C18	0.4 (4)
O1—Cd1—N1—C21	−166.58 (17)	Cd1—N1—C17—C18	174.5 (2)
O5—Cd1—N1—C21	−81.24 (19)	N1—C17—C18—C19	−0.1 (4)
O3—Cd1—N1—C21	96.97 (17)	C17—C18—C19—C20	−0.3 (4)
N4—Cd1—N1—C21	−5.98 (16)	C18—C19—C20—C21	0.5 (4)
O4—Cd1—N1—C21	57.2 (2)	C18—C19—C20—C22	−179.8 (2)
C9—Cd1—N1—C21	82.12 (18)	C17—N1—C21—C20	−0.2 (4)
O1—Cd1—N4—C30	−134.6 (2)	Cd1—N1—C21—C20	−174.77 (18)
O5—Cd1—N4—C30	−48.8 (2)	C17—N1—C21—C27	179.5 (2)
O3—Cd1—N4—C30	88.9 (2)	Cd1—N1—C21—C27	4.9 (3)
N1—Cd1—N4—C30	−179.8 (2)	C19—C20—C21—N1	−0.3 (4)
O4—Cd1—N4—C30	36.3 (2)	C22—C20—C21—N1	−180.0 (2)
C9—Cd1—N4—C30	62.1 (2)	C19—C20—C21—C27	−179.9 (2)
O1—Cd1—N4—C27	51.8 (3)	C22—C20—C21—C27	0.4 (4)
O5—Cd1—N4—C27	137.60 (18)	C23—N2—C22—C25	0.6 (4)
O3—Cd1—N4—C27	−84.68 (19)	C23—N2—C22—C20	−179.6 (2)
N1—Cd1—N4—C27	6.57 (17)	C19—C20—C22—N2	0.0 (4)
O4—Cd1—N4—C27	−137.30 (18)	C21—C20—C22—N2	179.7 (2)
C9—Cd1—N4—C27	−111.47 (18)	C19—C20—C22—C25	179.8 (2)
Cd1—O1—C1—O2	−14.5 (4)	C21—C20—C22—C25	−0.5 (4)
Cd1—O1—C1—C2	164.98 (18)	C22—N2—C23—C24	0.0 (4)
O2—C1—C2—C3	179.9 (3)	C25—N3—C24—C23	0.3 (4)
O1—C1—C2—C3	0.4 (4)	N2—C23—C24—N3	−0.5 (5)
O2—C1—C2—C7	2.2 (4)	C24—N3—C25—C22	0.4 (4)
O1—C1—C2—C7	−177.3 (3)	C24—N3—C25—C26	179.3 (2)
C7—C2—C3—C4	0.7 (4)	N2—C22—C25—N3	−0.9 (4)
C1—C2—C3—C4	−177.0 (2)	C20—C22—C25—N3	179.4 (2)
C2—C3—C4—C5	−0.6 (4)	N2—C22—C25—C26	−179.8 (2)
C3—C4—C5—C6	−0.3 (4)	C20—C22—C25—C26	0.5 (4)
C3—C4—C5—C8	178.3 (2)	N3—C25—C26—C28	0.5 (4)
C4—C5—C6—C7	1.1 (4)	C22—C25—C26—C28	179.5 (3)
C8—C5—C6—C7	−177.5 (3)	N3—C25—C26—C27	−179.3 (2)
C5—C6—C7—C2	−1.0 (5)	C22—C25—C26—C27	−0.3 (4)
C3—C2—C7—C6	0.1 (4)	C30—N4—C27—C26	0.5 (4)
C1—C2—C7—C6	177.9 (3)	Cd1—N4—C27—C26	174.48 (19)
C6—C5—C8—C8^i^	0.9 (5)	C30—N4—C27—C21	179.3 (2)
C4—C5—C8—C8^i^	−177.6 (3)	Cd1—N4—C27—C21	−6.7 (3)
Cd1—O3—C9—O4	3.2 (3)	C28—C26—C27—N4	−0.8 (4)
Cd1—O3—C9—C10	−175.37 (19)	C25—C26—C27—N4	179.0 (2)
Cd1—O4—C9—O3	−3.0 (2)	C28—C26—C27—C21	−179.6 (2)
Cd1—O4—C9—C10	175.6 (2)	C25—C26—C27—C21	0.2 (4)
O1—Cd1—C9—O3	−65.23 (16)	N1—C21—C27—N4	1.2 (3)
O5—Cd1—C9—O3	−159.83 (15)	C20—C21—C27—N4	−179.1 (2)
N4—Cd1—C9—O3	106.57 (16)	N1—C21—C27—C26	−179.9 (2)
N1—Cd1—C9—O3	33.98 (18)	C20—C21—C27—C26	−0.3 (4)
O4—Cd1—C9—O3	177.0 (2)	C27—C26—C28—C29	0.8 (4)
O1—Cd1—C9—O4	117.82 (14)	C25—C26—C28—C29	−179.0 (3)
O5—Cd1—C9—O4	23.22 (16)	C26—C28—C29—C30	−0.6 (5)
O3—Cd1—C9—O4	−177.0 (2)	C27—N4—C30—C29	−0.2 (4)
N4—Cd1—C9—O4	−70.38 (15)	Cd1—N4—C30—C29	−173.6 (2)
N1—Cd1—C9—O4	−142.97 (13)	C28—C29—C30—N4	0.3 (5)
O1—Cd1—C9—C10	−23.3 (19)	C33—N5—C31—O6	179.8 (4)
O5—Cd1—C9—C10	−117.9 (19)	C32—N5—C31—O6	−0.2 (3)
O3—Cd1—C9—C10	42.0 (18)		

Symmetry codes: (i) −*x*+3, −*y*, −*z*+1; (ii) −*x*+3, −*y*+2, −*z*.

### Hydrogen-bond geometry (Å, °)

**Table:**

*D*—H···*A*	*D*—H	H···*A*	*D*···*A*	*D*—H···*A*
O5—H5A···O2	0.84 (1)	1.85 (1)	2.655 (3)	158 (3)
O5—H5B···O4^iii^	0.84 (1)	1.88 (1)	2.713 (3)	169 (3)

Symmetry codes: (iii) −*x*+2, −*y*+1, −*z*.
